# Nitroglycerin Tolerance in Caveolin-1 Deficient Mice

**DOI:** 10.1371/journal.pone.0104101

**Published:** 2014-08-26

**Authors:** Mao Mao, Sudhahar Varadarajan, Tohru Fukai, Farnaz R. Bakhshi, Olga Chernaya, Samuel C. Dudley, Richard D. Minshall, Marcelo G. Bonini

**Affiliations:** 1 Department of Medicine-Section of Cardiology, University of Illinois at Chicago, Chicago, Illinois, United States of America; 2 Department of Pharmacology, University of Illinois at Chicago, Chicago, Illinois, United States of America; 3 Department of Anesthesiology, University of Illinois at Chicago, Chicago, Illinois, United States of America; 4 Department of Pathology, University of Illinois at Chicago, Chicago, Illinois, United States of America; University of Kansas Medical Center, United States of America

## Abstract

Nitrate tolerance developed after persistent nitroglycerin (GTN) exposure limits its clinical utility. Previously, we have shown that the vasodilatory action of GTN is dependent on endothelial nitric oxide synthase (eNOS/NOS3) activity. Caveolin-1 (Cav-1) is known to interact with NOS3 on the cytoplasmic side of cholesterol-enriched plasma membrane microdomains (caveolae) and to inhibit NOS3 activity. Loss of Cav-1 expression results in NOS3 hyperactivation and uncoupling, converting NOS3 into a source of superoxide radicals, peroxynitrite, and oxidative stress. Therefore, we hypothesized that nitrate tolerance induced by persistent GTN treatment results from NOS3 dysfunction and vascular toxicity. Exposure to GTN for 48–72 h resulted in nitrosation and depletion (>50%) of Cav-1, NOS3 uncoupling as measured by an increase in peroxynitrite production (>100%), and endothelial toxicity in cultured cells. In the Cav-1 deficient mice, NOS3 dysfunction was accompanied by GTN tolerance (>50% dilation inhibition at low GTN concentrations). In conclusion, GTN tolerance results from Cav-1 modification and depletion by GTN that causes persistent NOS3 activation and uncoupling, preventing it from participating in GTN-medicated vasodilation.

## Introduction

Nitrate tolerance is a well-characterized form of endothelial dysfunction that develops when cells, animals and patients are uninterruptedly exposed to nitrate vasodilators over extended periods of time [1], [2]. Recent studies on the prototype compound, nitroglycerin (GTN), have indicated that NO derived from endothelial nitric oxide synthase (eNOS/NOS3) activity is required for the vasodilator effect of GTN when used in pharmacological concentrations [3], [4]. As the etiology of nitrate tolerance remains controversial and poorly defined, we examined the possibility that NOS3 dysfunction contributes to the refractoriness of the endothelium to further stimulation with GTN (tolerance) or physiologic vasodilators that operate through NOS3 activation (cross-tolerance). This study was further motivated by the observation that caveolin-1 mice are normally refractory to low concentration GTN-induced vasodilation.

One cause of NOS dysfunction is persistent activation with cofactor depletion that lead to NOS uncoupling. Caveolin-1 (Cav-1) is an essential structural component of cholesterol-enriched lipid rafts known as caveolae [5], [6] which is known to play an important role in the regulation of cellular signaling by directly binding to numerous proteins through its caveolin-scaffolding domain (CSD) [7]–[12]. NOS3, perhaps the most studied CSD binding partner [13], [14], is known to be sequestered and inhibited by Cav-1 at the plasma membrane under basal conditions. Cav-1 binding prevents NOS3 dysfunction provoked by oxidative stress [15]–[18]. Loss of Cav-1 has been shown to cause aberrant angiogenesis [19] and endothelial dysfunction [20], which may be due to NOS3 hyperactivation and oxidative stress. Ablation of NOS3 in Cav-1 knockout mice has been shown to attenuate many of detrimental effects of Cav-1 deficiency confirming the fundamental role of Cav-1 in limiting the destructive potential of unfettered NOS3 activation [21], [22] as NOS3 can be rapidly converted into a source of superoxide radicals, and as demonstrated here, peroxynitrite.

Since functional NOS3 is required to transduce signaling initiated by GTN and Cav-1 is necessary to maintain functional NOS3, we hypothesized that GTN tolerance may result from Cav-1 depletion leading to NOS3 dsfunction.

## Results

### GTN tolerance in caveolin-1 knockout mice

**Figure 1:**
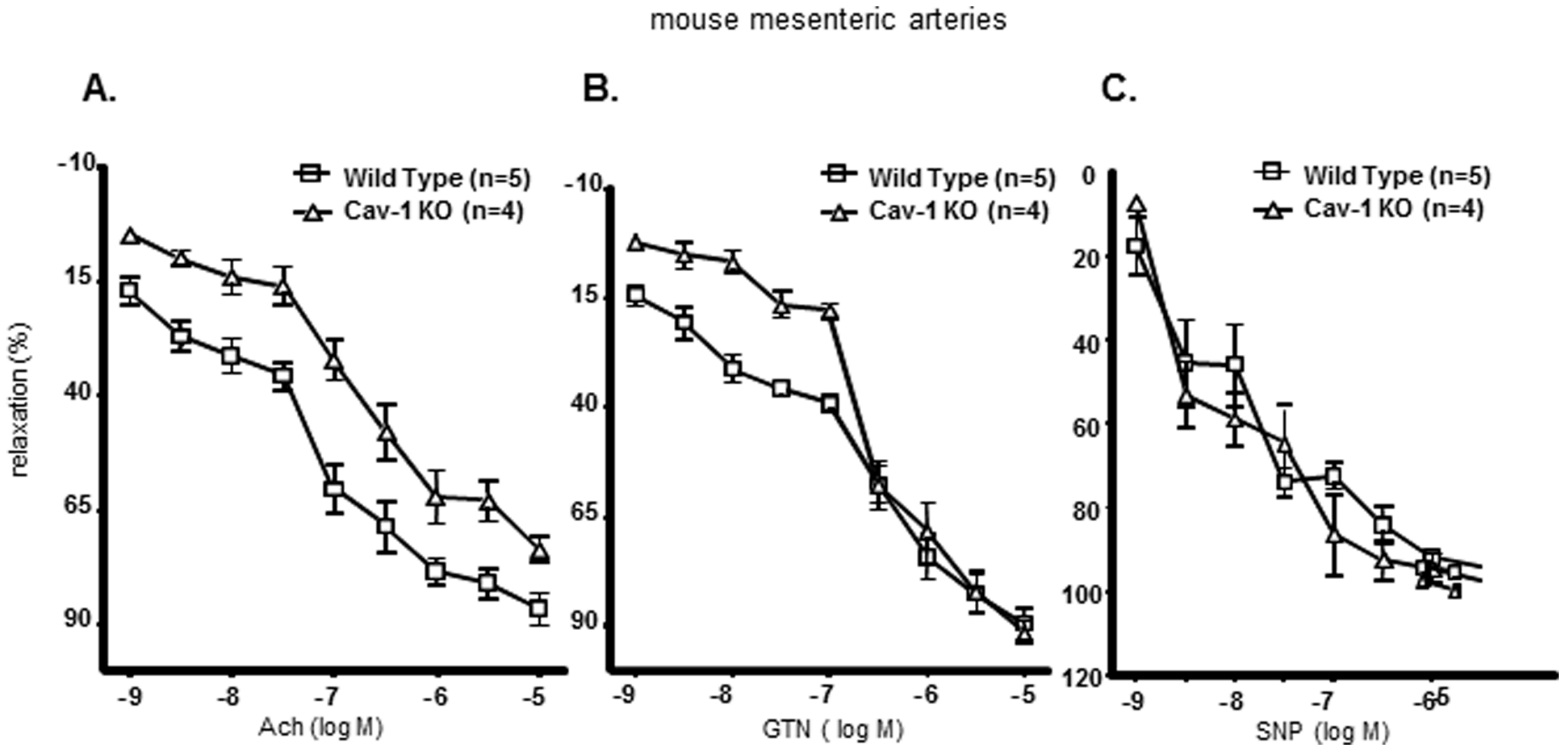
Caveolin-1 knockout mice are tolerant to GTN. Vasoreactivity experiments were performed in WT and Cav-1 -/- mouse mesenteric arteries. Results revealed that Cav-1 KO mice are resistant to both Acetylcholine (Ach, left panel) and low concentrations of GTN (middle panel). This indicates resistance is not due to defective signaling downstream of NO as shown by the normal responses of Cav-1 KO to sodium nitroprusside (SNP, right panel), a direct NO donor.

Cav1-KO mice showed marked tolerance to GTN used in pharmacologic concentrations (<100 nM). The concentration-response curve of the Cav-1^-/-^ mesenteric arteries to the physiologic vasodilator acetylcholine (Ach) was attenuated (curve shifted upwards and to the right) (**Fig. 1A**) compared to wild-type mouse mesenteric arteries. Thus, Cav-1^-/-^ mice exhibit tolerance across the entire tested concentration range of Ach, consistent with the hypothesis that Cav-1 depletion dampens the endothelial response to vasodilators that are dependent on NOS3 activity. Furthermore, mice devoid of Cav-1 were resistant to GTN at pharmacologic concentrations (<100 nM) where GTN effects were previously shown to be dependent on NOS function [3] (**Fig. 1B**). As shown in **Fig. 1C**, the concentration-response relationship of Cav-1^-/-^ mesenteric arteries to sodium nitroprusside (SNP) was indistinguishable from wild type mesenteric arteries responses, indicating (i) NOS fails to support NO production from Ach and low-concentration GTN in Cav-1^-/-^ mesenteric arteries and (ii) vasodilatory signaling downstream of NO is preserved in Cav-1^-/-^ mesenteric arteries. Previously, we showed that signaling responses to GTN in rat aortic rings and mice mesenteric arteries were qualitatively and quantitatively comparable [4]. Therefore, for the studies shown in Fig. 1 mesenteric arteries (a type of resistance vessels significantly impacting overall blood pressure) were chosen.

### Continuous GTN-exposure leads to Cav-1 degradation, NOS3 hyperactivation and dysfunction

**Figure 2:**
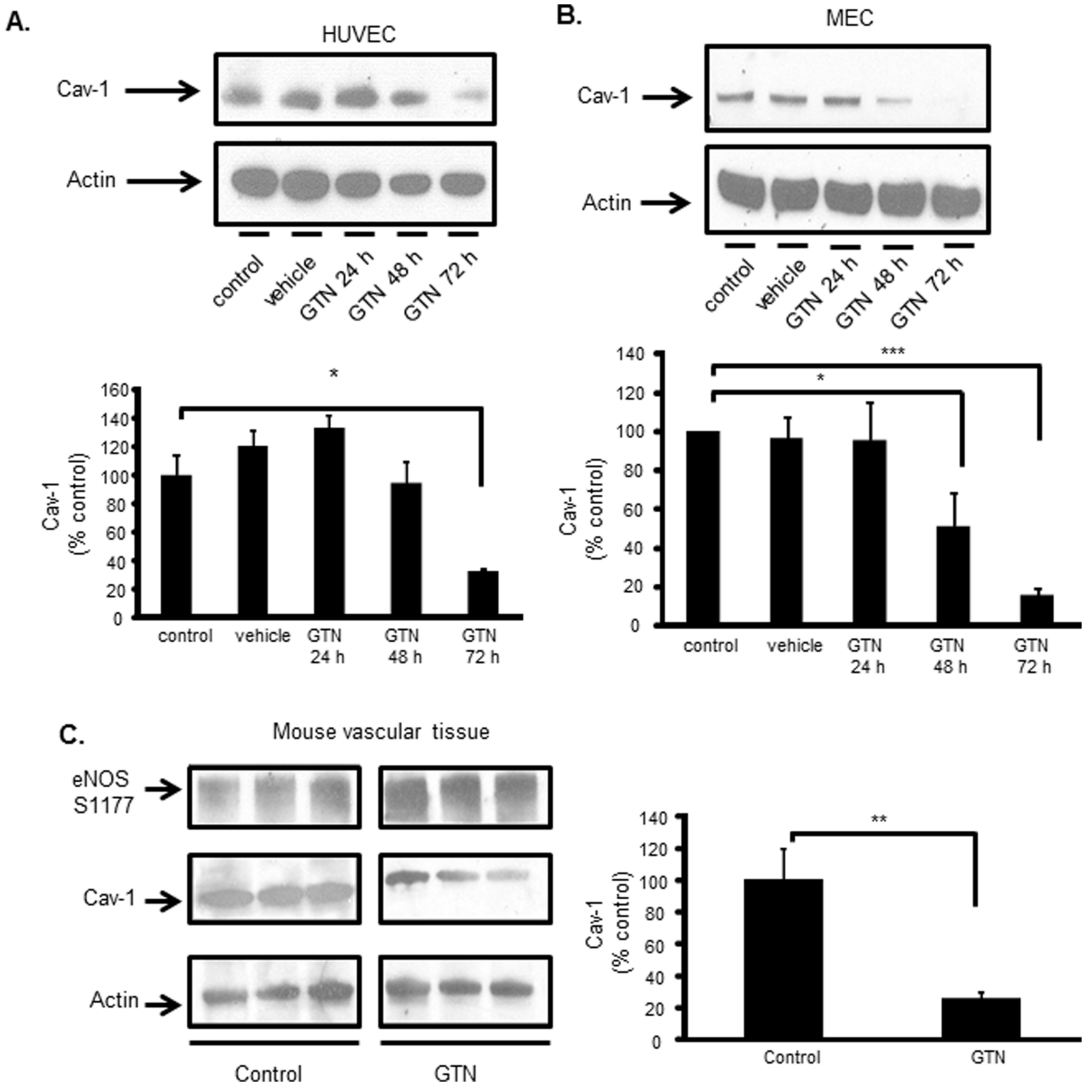
Continuous GTN-exposure leads to Cav-1 loss, NOS3 hyperactivation and dysfunction. Representative western blot image of at least three independent experiments showing GTN-induced cav-1 depletion in (A) human umbilical vein endothelial cell (HUVEC) and (B) primary mouse lung microvascular endothelial cells (MEC). Cells were treated with 20 µM GTN or vehicle control of indicated time. Cav-1 levels were examined by western blot. (C) Representative western blot of GTN-induction of cav-1 depletion paralleled by NOS3 activation in mouse aorta. Mice were exposed to GTN in the form of ointment at 2% concentration continuously for 72 h. Controls were exposed to vehicle-white petrolatum base paste. Bar graph shows the relative amounts of cav-1 compared with control group (mean ± SD), n = 3, * *p*<0.05, ** *p*<0.01, *** *p*<0.001

To test whether GTN could directly impact Cav-1 expression and NOS3 function via regulation of Cav-1 levels in the endothelium, human umbilical vein endothelial cells (HUVEC), and mouse endothelial cells (MEC) were continuously exposed to GTN for 24, 48 and 72 h. As shown in **Fig. 2A/B**, Cav-1 levels decreased between 48 and 72 h after initiating GTN exposure in human and mouse endothelial cell cultures. While in human endothelial cells maximum Cav-1 depletion by GTN was noted at 72 h, Cav-1 levels were significantly reduced at 48 h in mouse endothelial cells. To assess the *in vivo* relevance of these findings, mice were treated with GTN daily for three days by dermal application of 2% GTN ointment (200 mg/Kg) every 8 h to the shaved mouse skin (scapula). As shown in **Fig. 2C**, Cav-1 levels were markedly reduced in the aorta of mice treated with GTN for 72 h. Furthermore, loss of Cav-1 was paralleled by an increase in NOS3 active site Ser1177 phosphorylation (**Fig 2C**) indicating eNOS activation.

**Figure 3:**
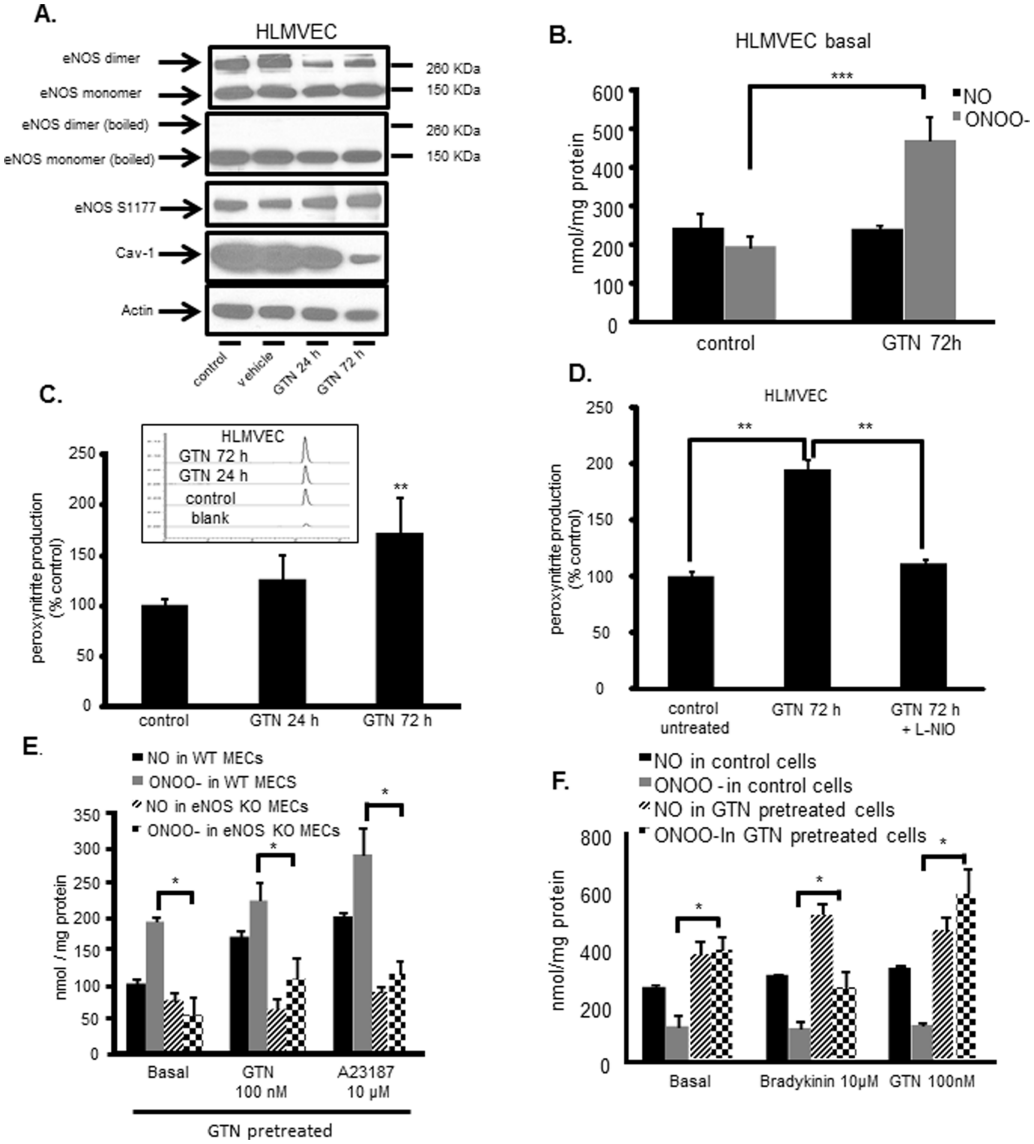
NOS3 dysfunction and peroxynitrite production in GTN exposed endothelial cells. (A) Exposure of human lung microvascular endothelial cells (HLMVEC) to the GTN alters NOS3 dimer/monomer distribution and provokes NOS3 Ser1177 phosphorylation. Cell lysates were subjected to low-temperature SDS-PAGE (LT-PAGE) to assess NOS3 dimer/monomer ration. Representative Western blots show endothelial nitric oxide synthase dimer/monomer in unprocessed (top) and denatured samples boiled in presence of 2-mercaptoethanol (second top). (B) Representative chemiluminescence measurement of basal NO and peroxynitrite production in GTN tolerant HLMVECs. Cells were treated with 20 µM GTN for 72 h. n = 6, *** *p*<0.001 (C) Representative peroxynitrite measurement by coumarin-7-boronic acid (CBA), a specific fluorescent peroxynitrite probe. Primary mouse endothelial cells were pretreated with GTN and then incubated with 10 µM CBA for 1 hour. Measurement was performed using HPLC, insert shows an example of typical fluorescence signals, n = 4, ** *p*<0.01 (D) L-NIO inhibition of peroxynitrite in tolerant cells. HLMVEC were treated with GTN for 72 h and pre-incubated with NOS3 inhibitor L-NIO (50 µM) for 1 h in serum free media before measurement. n = 3, ** *p<*0.01 (E) NO and peroxynitrite production in WT and NOS3 KO MECs are measured basally and under stimulation of either GTN (100 nM) or NOS3 activator A23187 (10 µM). Results showing NOS3 KO MECs were incapable of responding to GTN and do not produce peroxynitrite after chronic exposure to GTN, indicating NOS3 is the effector of GTN action and toxicity. n = 6, * *p*<0.05 (F) Stimulation of mouse endothelial cells (MECs) with activators of NOS3 (vasodilatory agonists) in cells chronically exposed to GTN (20 µM, 72 h) induced peroxynitrite production, supporting the concept that GTN poisoning converts a vasodilating response into a vasocontricting behavior, elicited by NOS3 dysregulation via ONOO^−^. n = 4–6, * *p*<0.05, ** *p*<0.01

Further investigation of the GTN effects on eNOS showed that GTN-exposure of human endothelial cells leads to the accumulation of NOS3 in its monomeric form (**Fig. 3A**). NOS3 monomers are thought to represent a highly dysfunctional form of the enzyme which generates superoxide radical anion [23], [24]. In the presence of NO, superoxide is rapidly converted to the powerful oxidant peroxynitrite [25]. Thus, we next examined whether exposing cells to GTN resulted in NOS dysfunction (i.e. increased peroxynitrite production in lieu of NO under basal and stimulated conditions). **Fig. 3B/C** shows that GTN increased peroxynitrite production by HLMVEC in a time-dependent manner. This was shown in two different ways: by the chemiluminescence based analysis of NO_2_^−^ accumulation in media in absence and presence of tempol (**Fig. 3B**) and through the use of the peroxynitrite biosensor coumarin-7-boronate (CBA, **Fig. 3C**) [26]–[29]. The source of peroxynitrite production was confirmed to be NOS3 in experiments shown in **Fig. 3D and E**, where L-NIO or the use of NOS3^−/−^ endothelial cells were shown to effectively dampen peroxynitrite production in cells exposed to GTN. Moreover, GTN pretreatment caused endothelial cells to respond abnormally not only to GTN stimulation, but also to bradykinin (a physiologic vasodilator).

We also found that GTN-induced peroxynitrite production was not dependent on NADPH oxidase (**Fig. S1**). Both NO and peroxynitrite were reduced in gp91phox^-/-^ cells exposed to GTN in comparison to wild-type suggesting that NADPH oxidase may indirectly contribute to peroxynitrite production by increasing NOS3 activation and uncoupling. If otherwise NADPH oxidase had direct participation in peroxynitrite production by generating O_2_^•^-, we would have expected to observe an increase in NO paralleled by a decrease in peroxynitrite formation in gp91phox^−/−^ cells.

### GTN provokes the proteolytic degradation of Cav-1

**Figure 4:**
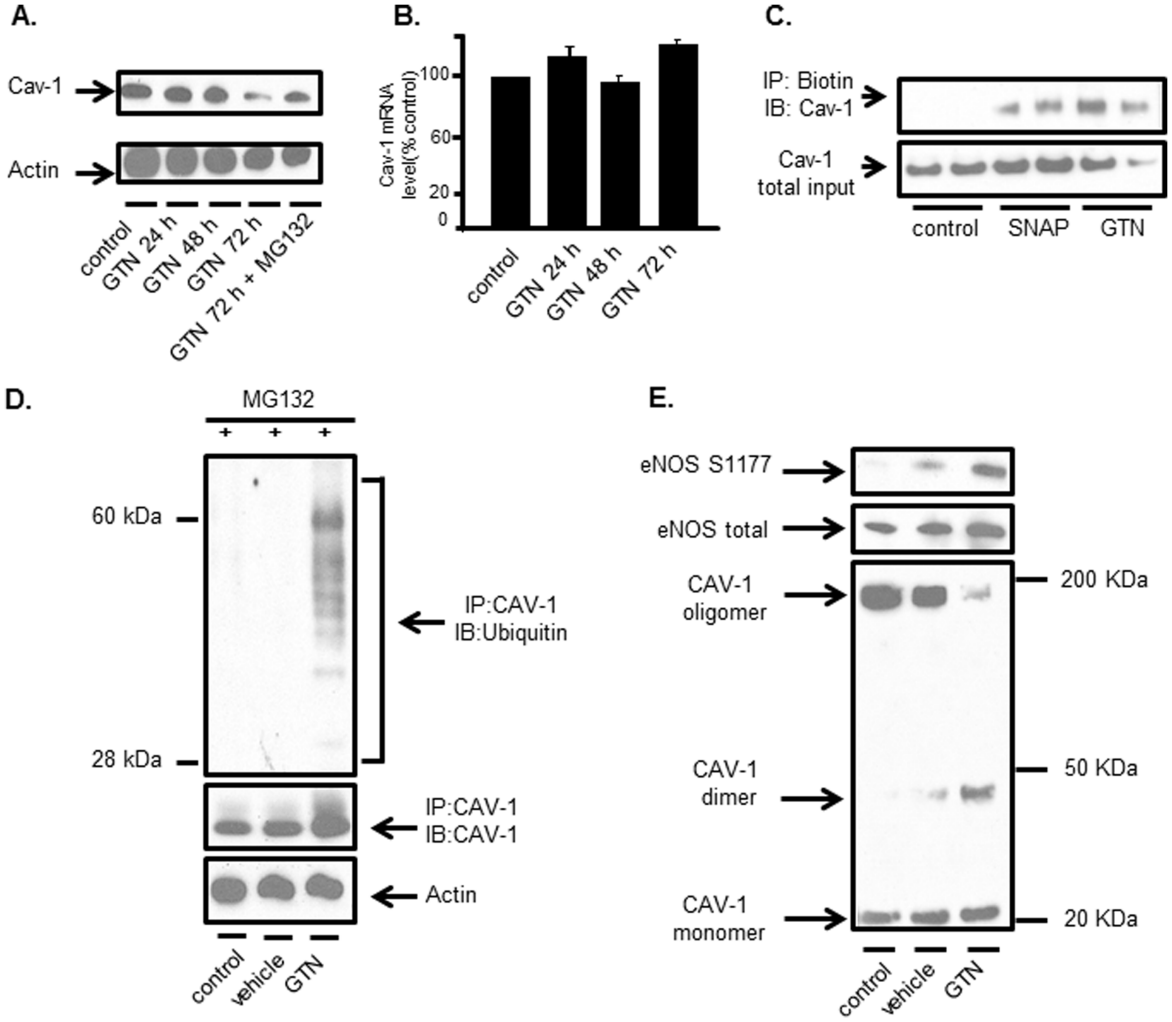
GTN provokes the proteolytic degradation of Cav-1. (A) Partial restoration of Cav-1 by proteasome inhibitor. MECs were treated with GTN (20 µM) for indicated time (24, 48 and 72 h). MG132 (5 µM) was added to the media and incubated over night (12 h) before lysing the cells. Cav-1 and Actin levels were analyzed by western blot. Results are representative of four independent experiments. (B) mRNA level (n = 3, mean ± SD) of cav-1 in MECs treated with GTN for 0, 24, 48 and 72 hours measured by nested PCR. GAPDH was used as internal standard for quantification. (C) Biotin switch assay of Cav-1 nitrosation. WT MECs were exposed to the nitrosation reagent, S-Nitroso-N-Acetylpenicillamine (SNAP, 1 mM) for 1 h or GTN (20 µM) for 72 h. S-nitrosated proteins were labeled with Biotin and pull-down with Streptavidin conjugated agarose beads. Whole cell lysates and eluted samples were analyzed for Cav-1 in western blot. Images are representative of three independent experiments. (D) Ubiquitination of immunoprecipitated Cav-1 in MECs. Cells were treated with GTN or vehicle for 72 hours, and incubated with MG132 (25 µM) 4 hours prior to lysis and immunoprecipitation. Ubiquitination levels in immunoprecipitated Cav-1 were examined using western blot. Actin and Cav-1 were analyzed as references of total input. Results are representative of three independent experiments. (E) Cav-1 oligomer/monomer distribution in tolerant cells assessed by non-reducing SDS-Page. MECs were treated with GTN or vehicle for 72 h. Cells lysates were subjected to native SDS-PAGE without addition of reducing reagent and boiling. Results shown are representative of three independent experiments.

After showing that GTN depletes Cav-1 in endothelial cells, further experiments were performed to determine the mechanism of GTN-induced Cav-1 loss. We found that MG132, a proteasomal inhibitor, when added to the cell culture media after the initiation of GTN treatment, prevented loss of Cav-1 indicating that GTN provokes dynamic Cav-1 proteolytic degradation (**Fig. 4A**). Confirmatory experiments showed that Cav-1 expression at the transcriptional level (mRNA) was not altered by GTN, which is consistent with the hypothesis that GTN-induced Cav-1 loss involves an increased rate of proteasomal degradation (**Fig. 4B**). To further gauge the mechanisms of GTN-induced Cav-1 loss, cells were treated with GTN continuously for different amounts of time in the absence or presence of proteasomal inhibitor MG132. To avoid toxicity, MG132 was added to culture media 2-4 h prior to the lysis of the cells. After immunoprecipitation, Western blot analysis of immunopurified Cav-1 demonstrated that Cav-1 was S-nitrosated solely in the samples that had been exposed to GTN or an alternative NO-donor S-nitroso-penicillamine (SNAP) (**Fig. 4C**). Previously, we have shown that Cav-1 Cys156 nitrosation promotes Cav-1 monomerization, polyubiquitination on Lys86, and degradation [30]. In the case of exposure to GTN, Cav-1 S-nitrosation paralleled the accumulation of polyubiquitinated Cav-1, a hallmark protein targeting for proteasomal degradation (**Fig. 4D**). Exposure of endothelial cells to GTN caused Cav-1 S-nitrosation, monomerization, ubiquitination and degradation (**Fig. 4E**). In accordance with previously published results [30] these are sequential steps by which S-nitrosation of Cav-1 conduces to the loss of the protein by proteasomal degradation. As shown in **Fig. 4E**, Cav-1 monomerization and degradation paralleled NOS3 activation as assessed by phosphorylation on Ser1177, temporally linking Cav-1 loss to increased NOS3 activity.

### Caveolin-1 knockdown recapitulates NOS3-dysfunction and leads to oxidative stress observed in nitrate tolerance

**Figure 5:**
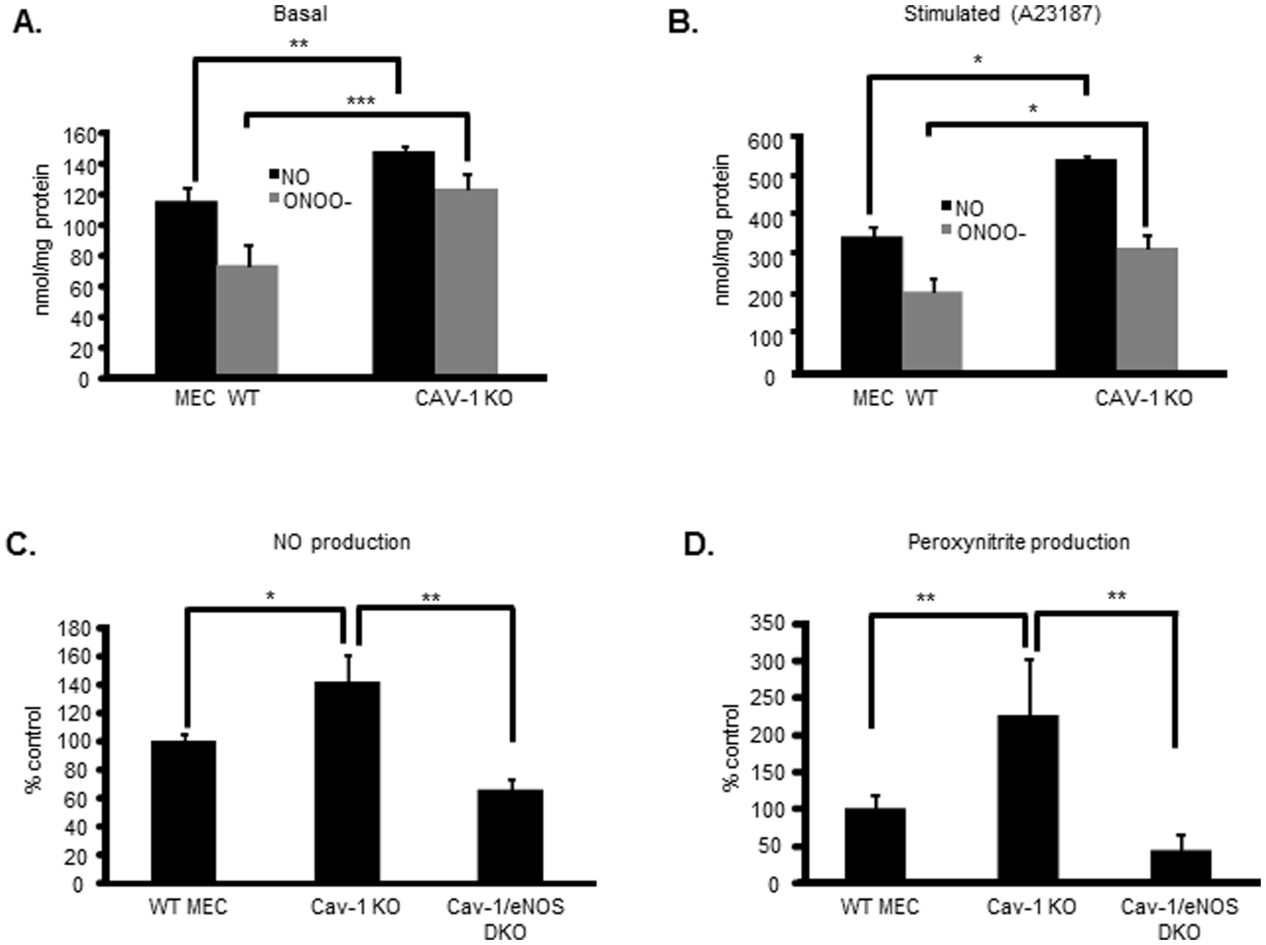
Caveolin-1 knockdown recapitulate NOS3 dysfunction and leads to nitrate tolerance. (A) Representative measurement of basal NO and peroxynitrite level in WT and Cav-1 KO MECs. n = 6, ** *p<*0.01, *** *p*<0.001 (B) Representative measurement of NO and peroxynitrite in WT and Cav-1 KO MEC stimulated with A23187 (10 µM) for 1 h. (C) Basal NO production in WT, Cav-1 knockout and Cav-1/NOS3 double knockout (DKO) MECs, n = 3 to 5, * p<0.05, ** *p*<0.01. (D) Basal Peroxynitrite production in WT, Cav-1 knockout and Cav-1/NOS3 double knockout MECs, n = 3 to 5, ** *p*<0.01.

If Cav-1 modification and degradation led to NOS3 uncoupling and nitrate tolerance, then Cav-1 knockout endothelial cells might be expected to be inherently under oxidative stress that characterizes tolerant endothelial cells. As shown in **Fig. 5A and 5B**, mouse endothelial cells depleted of Cav-1 produced markedly greater amount of peroxynitrite in comparison to wild type controls. Stimulation of Cav-1^−/−^ endothelial cells with the NOS3 activator calcium ionophore (A23187/ionomycin) increased both NO and peroxynitrite production. To confirm dysregulated NOS3 is the source of peroxynitrite, we utilized endothelial cells recovered from Cav-1/NOS3 double-KO mice. In these cells, NO and peroxynitrite production at baseline were lower than those measured in control cells at baseline (**Fig. 5 C**) or after A23187 stimulation (**Fig. 5 D**). These results confirmed that NOS3 dysfunction sustains peroxynitrite production in Cav-1^−/−^ endothelial cells. These results also indicated that NOS3 is a significant source of oxidants in cells devoid of Cav-1 and because GTN depletes Cav-1, NOS3 contributes to promote GTN-induced oxidative stress in tolerance.

**Figure 6:**
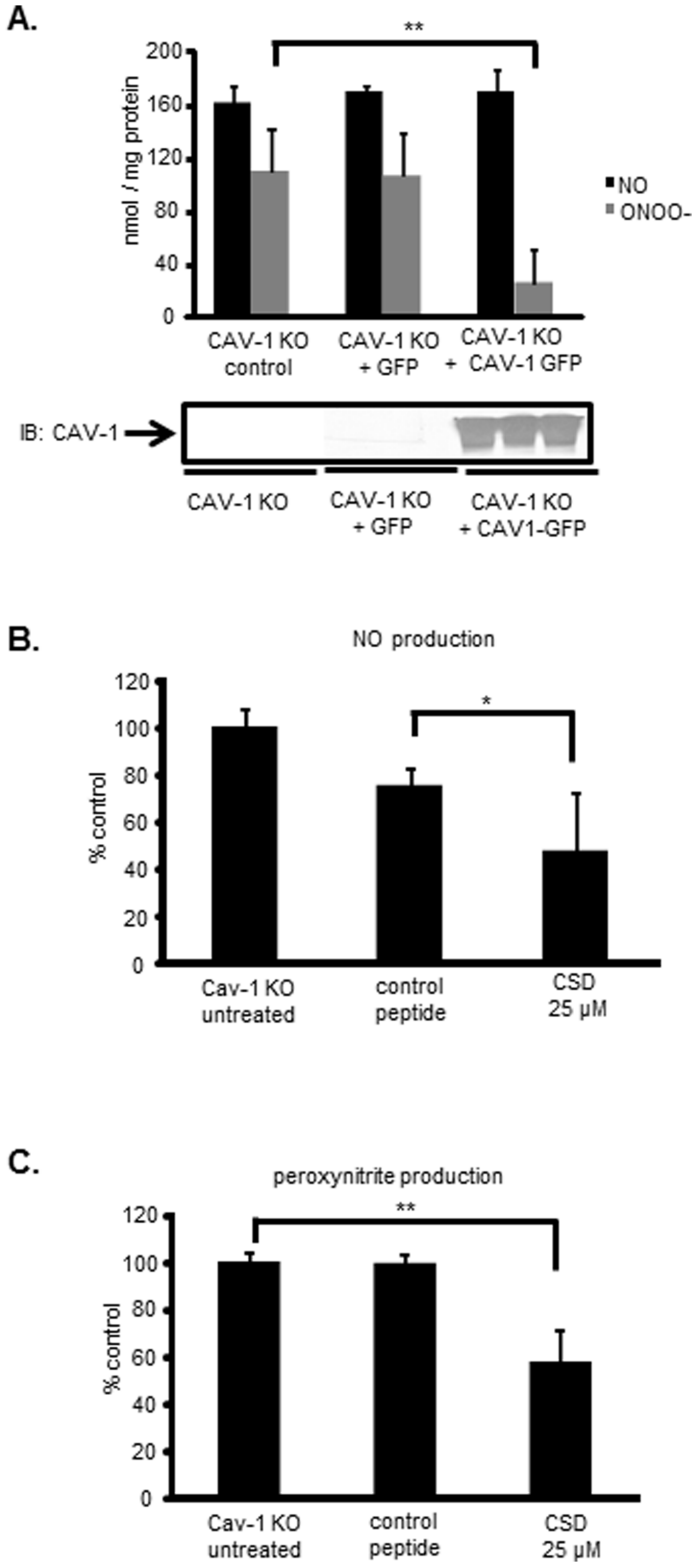
Expression of Caveolin-1 in Cav-1 knockout mouse endothelial cells eliminates peroxynitrite production. (A) NO and peroxynitrite measurement in Cav-1 -/- mouse endothelial cells transfected with either empty vector (EGFP) or wild type Cav-1-GFP construct. The production of NO and peroxynitrite was normalized by protein concentration. n = 6, ** *p*<0.01. Cav-1 expression was analyzed and confirmed by western blot. (B) NO production in Cav-1 KO MECs treated with cell permeable caveolin-1 scafold domain (CSD, 25 µM) (C) Peroxynitrite production in Cav-1 KO MECs treated with cell permeable caveolin-1 scafold domain (CSD, 25 µM), n = 4, * *p*<0.05, ** *p*<0.01, results are representative of three independent experiments.

To confirm that NOS3 dysfunction secondary to Cav-1 loss contributes to uncoupled NOS3, we restored Cav-1 expression in Cav-1*^−/−^* cells. As shown in **Fig. 6A**, Cav-1 reconstitution dramatically reduced peroxynitrite production from Cav-1*^−/−^* cells. In addition, the cell permeable Cav-1 scaffold domain peptide (CSD) effectively reduced NOS3-derived NO (**Fig. 6B**) and peroxynitrite (**Fig. 6C**) production, consistent with previous reports that showed that scaffolding of NOS3 impedes dysfunctional NOS3 activity [31]. Differently from Cav-1 itself, the CSD peptide inhibited both peroxynitrite and NO production by endothelial cells suggesting that while CSD functions to inhibit NOS3, Cav-1 may have more complex regulatory effects on NOS3 activity than pure inhibition.

### GTN but not PETN causes Cav-1 downregulation, eNOS dysfunction and oxidative stress

**Figure 7:**
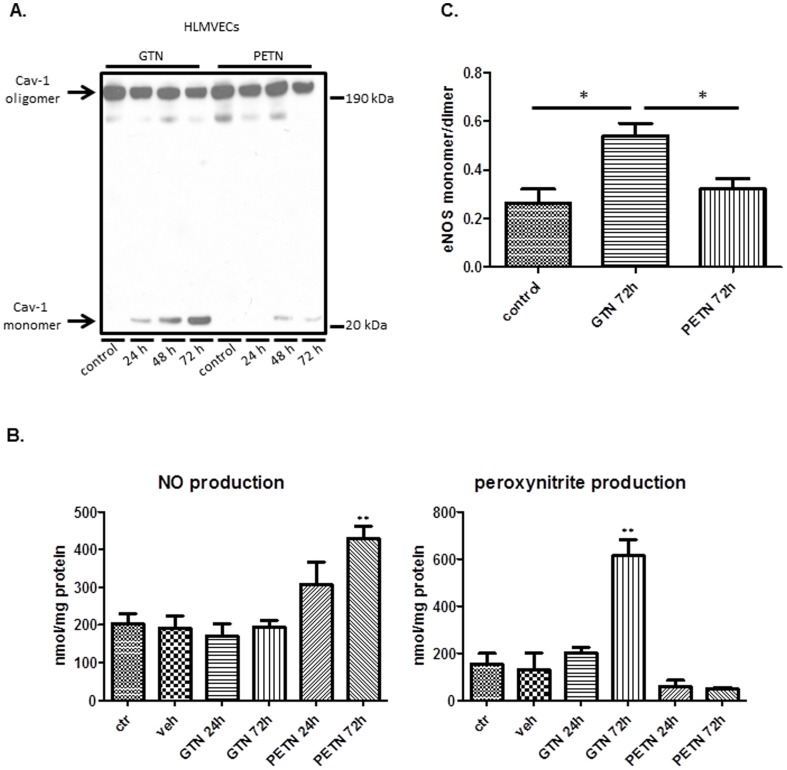
PETN has less adverse effect on Cav-1 and eNOS. (A) Representative western blot of Cav-1 oligomer/monomer distribution in HLMVECs exposed to GTN (20 µM) and PETN (10 µM) for indicated time (24, 48 and 72 h). (B) Nitric oxide (NO) and peroxynitrite (ONOO-) measurement in HLMVECs exposed to GTN and PETN for indicated time. ** *p*<0.01, n = 4–6 (C) Quantified eNOS monomer/dimer ratio in HLMVECs exposed to GTN and PETN for 72 h. * *p*<0.05, n = 5.

On comparing the effects of GTN on endothelial cells with those of PETN, an organic nitrate that does not cause tolerance [32]–[34], we observed that PETN preserved functional Cav-1 oligomers while GTN promoted Cav-1 accumulation in the degradation-prone monomeric form (**Fig. 7A**). Accumulation of monomeric Cav-1 was accompanied by an increase in monomeric over dimeric eNOS in the case of GTN but not when cells were exposed to PETN (**Fig. 7B**). Functionally, exposure to PETN increased NO outputs from HLMEC without increasing peroxynitrite formation in clear opposition to GTN that enhanced peroxynitrite without significant measurable effects on NO outputs. Collectively, these results are in agreement with the reported effects of PETN in alleviating hypertensive conditions without producing tolerance and indicate that Cav-1 depletion and eNOS dysfunction by GTN could, at least in part, account for the onset of nitrate tolerance.

## Discussion

Nitroglycerin (GTN) is used to treat heart failure, angina, and hypertension. It has been known for decades that chronic exposure to GTN and other nitrates rapidly induce tolerance, a well-characterized form of endothelium dysfunction that renders the vasculature refractory to vasodilator stimuli. Today, nitrate tolerance continues to be a clinically relevant problem in view of the many prescriptions written in the U. S. alone and the fact that GTN is widely used as an explosive, detonator, and propellant, thus leading to environmental contamination and casual exposure. The toxic effects of chronic exposure to GTN have been extensively documented in the past because of occupational exposures, but the mechanisms of toxicity, long-term consequences of exposure and potential transgenerational impacts have not been assessed in detail.

**Figure 8:**
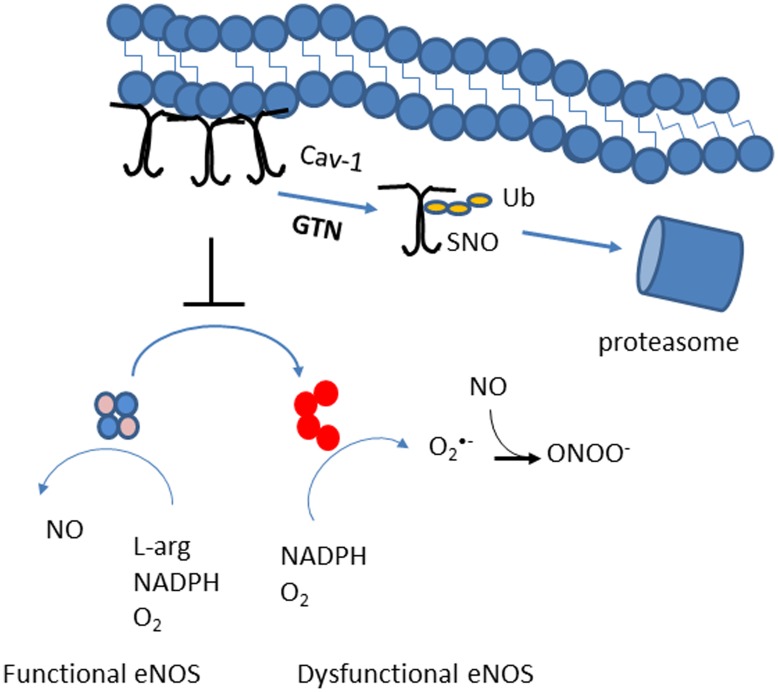
Schematic representation of GTN-induced Cav-1 degradation and NOS3 dysfunction. Figure summarizes main findings in this study and in [30] that together indicate that S-nitrosated Cav-1 is degraded by the proteasome promoting eNOS dysfunction and a transition from the NO synthase activity to that of a NADPH oxidase.

We show that GTN tolerance results from GTN-induced post translational modifications to Cav-1 protein that prompt Cav-1 degradation. Dampening of Cav-1 levels results in NOS3 dysfunction as assessed by multiple functional and structural parameters (**Figs. 4** **–** **6**). NOS3 turned incapable of adequately responding because of loss of Cav-1 fails to transduce stimuli of pharmacologic origin e.g. GTN or physiologic vasodilators, e.g. bradykynin and acetylcholine and by subsequent acute GTN administration (tolerance). As schematically represented in **Fig. 8** we found that despite significant Cav-1 protein loss (**Fig. 2A/B**), mRNA levels remained unaltered by GTN through the course of treatments (up to 72 h) (Fig. **4B**) consistent with the rapid recovery of vascular responses to GTN upon suspension of exposure. Importantly, all of the GTN-induced effects were recapitulated in the Cav-1 knockout cells demonstrating that Cav-1 depletion in a completely independent model results in the same effects of GTN treatment pertaining to the induction of NOS3 dysfunction, oxidative stress and GTN tolerance (**Fig. 1**, **4** **–** **6**). In experiments using NOS inhibitor (L-NIO) or genetic NOS3 knockout or double knockout, it became evident that peroxynitrite results from abnormal NOS3 function in the absence of Cav-1. Such conclusion is corroborated by the finding that reconstitution of Cav-1 expression or the use of mimetic peptides markedly dampened peroxynitrite production by Cav-1 KO cells (**Fig. 6**). In summary, we demonstrate that either continuous GTN treatment or Cav-1 knockout animals become: 1 - basally resistant to low concentration GTN, 2 - completely responsive to high concentration GTN and sodium nitroprusside (SNP), and 3 - have a blunted concentration-response to physiologic vasodilators. These findings support our contention that functional NOS3 mediates low concentration GTN-induced vasodilation (and responses to acetylcholine as demonstrated by others [35]), while bioactivation by other identified routes accounts for the immediate vascular effects of high GTN concentrations. Nevertheless, our observation regarding nitrate tolerance does not rule out the possibility of alternative mechanisms. Various studies indicate that oxidation of aldehyde dehydrogenase-2 (ALDH2), a redox-sensitive mitochondria resident enzyme, is potentially associated to both the bioactivation of GTN and the development of nitrate tolerance [36]–[39]. This is thought to be because GTN oxidizes the critical low-pKa thiolate moiety of ALDH2 disabling the enzyme to convert GTN into NO_2_^−^ and subsequently NO.

Importantly, on comparing the effects of PETN and GTN we observed that differently than GTN, PETN preserves Cav-1 and eNOS function. PETN is known to alleviate hypertensive conditions without causing tolerance. This observation further supports the concept that GTN causes tolerance, in part, by producing eNOS dysfunction possibly originating from Cav-1 depletion.

The current study provides a mechanism for nitrate tolerance. NOS3 dysfunction and oxidative stress that ensues following chronic GTN exposure are likely to accentuate endothelial dysfunction (leading to (cross) tolerance). These observations could be used to design strategies to prevent GTN tolerance, such as use of concomitant Cav-1 mimetic peptides or BH_4_ precursors, a cofactor lost in NOS uncoupling.

## Materials and Methods

### Chemicals and reagents

Nitroglycerin was from American Regent (Shirley, NY). Rabbit polyclonal anti-eNOS/NOS 3, mouse anti-phosphorylated NOS3 (Ser 1177) and mouse anti-Caveolin-1 antibodies were from BD Biosciences (Franklin Lakes, NJ). Rabbit polyclonal anti-ubiquitin was from Abcam (Cambridge, MA). Protein A/G agarose bead was from Santa Cruz (Santa Cruz, CA). Dynabeads was from (Invitrogen Dynal, Oslo, Norway). Coumarin-7-boronate was synthesized in Dr. Balaraman Kalyanaraman's lab at Medical College of Wisconsin (Milwaukee, WI). Caveolin-1 scafolding domain peptide (CSD) and scrambled control peptides were from Calbiochem (San Diego, CA). L-NIO was from Cayman Chemical (Ann Arbor, MI). S-Nitroso-N-acetylpenicillamine (SNAP) and 4-hydroxyl-Tempol were from Sigma Aldrich (St. Louis, MO). Nucleofector kit was from Lonza (Walkersville, MD). Cav-1-YFP and empty YFP vector were provided by Dr. Rich Minshall at University of Illinois at Chicago. All other reagents are analytical grade or better.

### Cell Cultures

Human microvascular endothelial cells (HMECs) and Human umbilical vein endothelial cells (HUVECs) were cultured in fully supplemented EBM-2 endothelial growth media with 10% FBS and 1% Antibiotics – Antimycotics (Sigma, St. Louis, MO) and used at passages 7–12. Primary mouse endothelial cells (MECs) were cultured at 37°C, 5% CO2 in corresponding endothelial media with supplements including growth factors, 10% FBS and 1% Antibiotics – Antimycotics. HMEC cells were from Cell Systems (Kirkland, WA). HUVEC were from Lonza (Alpharetta, GA).

### Western blot assays

Samples were lysed in RIPA buffer containing protein inhibitor cocktail (Roche's complete mini) and phosphatase inhibitors (20 mM NaF, 5 mM NaVO_3_, 1 mM NaVO_4_, 2.5 mM glycerol phosphate and 10 mM sodium pyrophosphate). Lysates were centrifuged at 13,000 rpm for 10 minutes and the supernatant was recovered. Protein concentration was measured using BCA method. Samples were then mixed with loading buffer and separated using 4–12% Bis-Tris pre-cast gradient mini gel from Invitrogen and blotted onto nitrocellulose membranes. After overnight blocking with 5% fat-free milk, specific primary and secondary antibodies were incubated with the membranes at the indicated dilutions and time. Bands were detected by chemiluminescence using ECL kit from (Pierce, Rockford, IL). Densitometry was performed using the software Image J from the National Institutes of Health (NIH).

### Low-Temperature SDS-PAGE analyses of NOS3 dimer/monomer

Cultured cells or tissues from animal were prepared in lysis buffer under non-denaturating conditions. Samples were loaded on to 4–12% polyacrylamide gels and subjected to electrophoresis. During electrophoresis process, buffers and gels were placed in ice-water bath and the whole apparatus was kept at 4°C. After tranfering the proteins on to nitrocellulose membrane, the monomer and dimer forms of NOS3 were detected by Western blot.

### Immunuoprecipitation of Cav-1

Serum starved mouse endothelial cells were treated with designated stimulus. After 15 minutes, media was removed. Cells were washed twice with TBS and lysed in lysis buffer containing protease and phosphatase inhibitors. After determining total protein concentration by BCA assay, 500 µg protein of each sample was incubated with 5 µg mouse anti-Cav-1 antibody overnight and then pulled down using 20 µl anti-mouse IgG Dynabeads, in some experiments protein A/G agarose beads from Santa Cruz were also used. For the analysis of ubiquitination, cells were pre-treated with MG-132 (Calbiochem) for indicated concentration and time. Ubiquitination of the samples were examined using a rabbit anti-ubiquitin antibody.

### RT-PCR analysis of Cav-1 mRNA level

Total cell RNA was extracted using Qiagen RNeasy kit (Qiagen, Valencia, CA) and reverse transcribed. 100 ng of cDNA template was used in each reaction. Nested was performed to determine expression of Cav-1 and GAPDH (housekeeping gene) using corresponding primers obtained from Santa Cruz Biotechnology (Santa Cruz, CA).

### Measurement of nitric oxide (NO) production by chemluminescence

Cells were washed twice with HBSS and incubated with serum free DMEM or HBSS at 37°C. The cells were then treated with agonists or inhibitors for indicated time. After treatment, media was collected and centrifuged shortly to remove floating cells. NO concentration in the culture media was assessed by measuring NO_2_^−^ accumulation using a Sievers 280i Nitric Oxide Analyzer (Sievers Instruments, Boulder, CO). NO release was assessed from acuminated NO_2_^−^ level in the media and reported as nmol NO per mg protein. For the measurement of peroxynitrite formation, parallel groups were incubated with and without Tempol. The difference between NO measurements from the two groups was used to present level of peroxynitrite. Qualitatively and quantitatively the chemiluminescent method of peroxynitrite assessment with the assistance of tempol was comparable to the validated CBA method. Additional data validating the method are presented in Fig. S2.

### Measurement of peroxynitrite production by Coumarin-7-boronate (CBA)

Cells were grown to full confluence in 100 mm dishes and treated with different reagents (vehicle or GTN) for indicated time. Cells were then washed twice with DPBS and incubated with media containing 20 µM CBA for 60 minutes. Media was collected and centrifuged at 3000 rpm for 5 min. Fluorescence analysis of the oxidation product 7-OH-coumarin (COH) was performed on a Beckman-Coulter HPLC system. Samples (20 µl) were separated on a Synergi-Fusion (250×4.6 mm, Phenomenex) using an isocratic elution with 35% v/v acetonitrile, at a flow rate of 1 ml/min. Fluorescence was measured using 350 nm (excitation) and 450 nm (emission). Authentic COH solution was injected onto the HPLC to make standard curve.

### Biotin switch assay

S-Nitrosylated protein was detected by the biotin-switch method as described previously [ref biotin switch] using Caymen Chemicals S-Nitrosylated Protein Detection Kit. Briefly, endothelial cells were treated with GTN for indicated time. MG132 (50 mM) was added to the media 1 h before cells were lysed. Biotin-switch method as described previously using an S-Nitrosylated Protein Detection Kit from Cayman Chemicals following the manufacturer's instructions. Samples were immunoprecipitated using streptavidin conjugated antibodies (Dynabeads M-280 Streptavidin from Invitrogen). Biotinylated Cav-1 was resolved by non-reducing SDS-polyacrylamide gel electrophoresis (PAGE), transferred and detected by immunoblotting with mouse anti-Cav-1 (1∶1000).

### Transient Cav-1-YFP transfection

Primary Caveolin-1 knockout mouse endothelial cells (Cav-1 KO MECs) were grown in media with supplements. At 100% confluence, cells were washed with PBS and harvested with 0.05% Trypsin-EDTA. Transfection was performed through electroporation using an Amaxa Nucleofector device following the manufacturer's protocol. For each reaction, 5×10^5^ cells were mixed with 10 ng cDNA and re-suspended in 100 µl Nucleofector reagent. After electroporation, cells were plated into 6-well plates and incubated (37°C, 5% CO_2_) for 48 hours. Basal NO and peroxynitrite was measured as NO_2_- accumulated in serum media accumulated for 2 h by chemiluminescence. After media sampling, cells were lysed for protein concentration measurement by BCA.

### Animal study

Animals utilized in this study were C75bl/6 male mice between 8–12 weeks of age. Control and Cav-1-/- mice on the C57bl/6 background were used for vasoreactivity assays. All procedures were approved by the National Institute of Environmental Health Sciences and University of Illinois at Chicago Institutional Animal Care and Utilization Committee (IACUC) under the policies, procedures and guidelines of National Institutes of Health. All surgical procedures were performed with deeply anesthetized mice (100 mg/Kg ketamine, 5 mg/Kg xylazine). Anesthesia was confirmed by lack of responsiveness to toe pinching. Euthanasia was performed by anesthetic overdose and ensured by cervical dislocation.

### Mesenteric artery dilation assay

Isometric tension of mesenteric resistance arteries were measured using wire myograph (Model 610 M, Danish Myo Technology, Denmark). Briefly, the first or second order branches of resistance arteries were isolated from mice mesenteric bed, cut into ∼2 mm segment and stored in cold Krebs Physiological Salt Solution (PSS) (119.0 mM NaCl, 25.0 mM NaHCO_3_, 4.6 mM KCl, 1.2 mM MgSO_4_, 1.8 mM CaCl_2_, 11.0 mM glucose) at pH 7.4. The vessel were mounted in between two hook using tungsten wire (25 µm in diameter) in organ chamber which containing Krebs PSS bubbled with gas mixture containing 5% CO_2_ and 95% O_2_. Basal tension was set on arteries stretched to L100, where L100 is defined as the circumference of the relaxed artery exposed to a transmural pressure of 100 mmHg and equilibrated for 1 hr. After equilibration, the arteries were exposed to high concentration of KCl (80 mM) and 10 µM norepinephrine for 2–3 min until reproducible maximal contractions occur. The α-adrenergic receptor agonist, phenylephrine was added to increase basal tension to 60% to 80% of maximal KCl contraction. Cumulative concentration (0.01–10 µM) of nitroglycerin (GTN) were added to the bathing solution every 5 min. At the end of the each experiment, cumulative concentration of sodium nitropruside (0.01–10 µM) was added to the bath to demonstrate the intact smooth muscle function. Results are expressed as percent relaxation of the phenylephrine-treated rings, with 100% relaxation representing basal tension.

### Statistical analysis

Data are expressed as mean ± SD unless otherwise mentioned. Statistical analysis was performed by Student's t test or two-way ANOVA analysis of variance.

## Supporting Information

Figure S1 **NADPH contributes to eNOS dysfunction in tolerance.** NO and peroxynitrite measurement in WT and gp91 phox KO mouse endothelial cells exposed to GTN (20 µM, 72 h), n = 3–6 ** *p*<0.01, *** *p*<0.001.
(TIF)

Figure S2 **Validation of the tempol-assisted method to assess peroxynitrite.** (**A**) Tempol does not alter authentic nitrite (NO_2_^−^) concentrations in aqueous solution at pH = 7.4. (**B**) Tempol does not influence the accumulation of NO_2_^−^ from the decomposition of an NO-donor (DEANO) in aqueous solution at pH = 7.4, incubations were performed at room temperature in PBS buffer (0.1 mM) for 1 h. (**C**) NO_2_^−^ accumulation from the decomposition of the peroxynitrite donor (SIN-1) is enhanced by tempol across a large range of tempol concentrations. This effect is attributed to the superoxide dismutase mimetic activity of tempol that spares NO from being quenched by O_2_^•−^ [40], [41].
(TIF)
